# The Role of Actin Turnover in Retrograde Actin Network Flow in Neuronal Growth Cones

**DOI:** 10.1371/journal.pone.0030959

**Published:** 2012-02-16

**Authors:** David Van Goor, Callen Hyland, Andrew W. Schaefer, Paul Forscher

**Affiliations:** Department of Molecular, Cellular, and Developmental Biology, Yale University, New Haven, Connecticut, United States of America; Northwestern University Feinberg School of Medicine, United States of America

## Abstract

The balance of actin filament polymerization and depolymerization maintains a steady state network treadmill in neuronal growth cones essential for motility and guidance. Here we have investigated the connection between depolymerization and treadmilling dynamics. We show that polymerization-competent barbed ends are concentrated at the leading edge and depolymerization is distributed throughout the peripheral domain. We found a high-to-low G-actin gradient between peripheral and central domains. Inhibiting turnover with jasplakinolide collapsed this gradient and lowered leading edge barbed end density. Ultrastructural analysis showed dramatic reduction of leading edge actin filament density and filament accumulation in central regions. Live cell imaging revealed that the leading edge retracted even as retrograde actin flow rate decreased exponentially. Inhibition of myosin II activity before jasplakinolide treatment lowered baseline retrograde flow rates and prevented leading edge retraction. Myosin II activity preferentially affected filopodial bundle disassembly distinct from the global effects of jasplakinolide on network turnover. We propose that growth cone retraction following turnover inhibition resulted from the persistence of myosin II contractility even as leading edge assembly rates decreased. The buildup of actin filaments in central regions combined with monomer depletion and reduced polymerization from barbed ends suggests a mechanism for the observed exponential decay in actin retrograde flow. Our results show that growth cone motility is critically dependent on continuous disassembly of the peripheral actin network.

## Introduction

Growth cone structure and motility emerge from the balance of actin filament polymerization, depolymerization, transport, and cross-linking into various network architectures. Motile actin structures continuously consume energy and cycle their components. As structure is continuously turned over, the system can respond rapidly to extracellular guidance cues; small changes affecting actin structure may lead to rapid changes in growth cone morphology and behavior. In the absence of changes in substrate or soluble guidance cues, actin filament polymerization, depolymerization, and transport maintain a steady-state balance where there is little change in position and growth cone morphology despite continuous retrograde actin flux. In such a system, perturbations of a single process can reveal information on the interdependency of the other processes in maintaining the steady-state balance.

**Figure 1:**
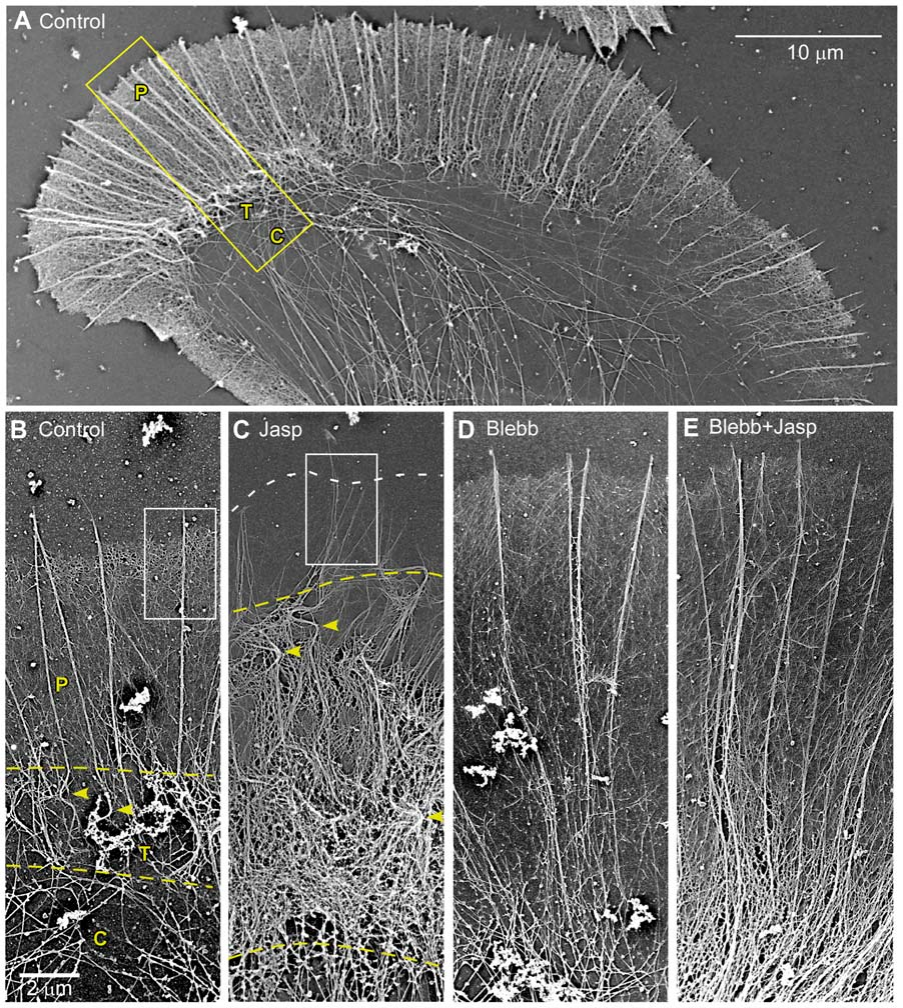
Jasplakinolide treatment results in a myosin II independent accumulation of actin filaments in the transition zone and a myosin II dependent depletion of veil actin in the periphery. (A) Electron micrograph of an untreated *A. californica* BCN growth cone showing domain organization. Growth cone electron micrographs of the peripheral domain and transition zones (typical ROI shown in A) of growth cones treated with vehicle for 6 minutes (B), 500 nM jasplakinolide for 6 minutes (C), 70 µM blebbistatin for 10 minutes (D), or 70 µM blebbistatin for 10 minutes followed by 70 µM blebbistatin and 500 nM jasplakinolide for 10 minutes (E). Following drug treatment, cells were live extracted and processed for platinum/palladium replica electron microscopy. Dashed yellow lines in panels A and B delineate the transition zone, where filopodia buckling occurs (examples marked with yellow arrowheads). Dashed white line in (C) denotes position of leading edge. The scale bar in D is 2 µm and applies to all panels.

Actin assembly at the leading edge produces filopodia and veil structures which are transported centripetally by a process called retrograde flow. Both leading edge polymerization and myosin II contractility contribute to retrograde actin movement. Addition of actin subunits between the polymerized actin array and the leading edge membrane pushes the entire network centripetally [1]. According to the dynamic network contraction model [2], myosin II localized in the transition zone (Fig. 1 and Fig. S1D–F) generates contractile force, which propagates through the mechanical continuum of the actin array and displaces the entire network. Retrograde flow rate is affected by myosin II activity. Flow rates decrease up to 50% in growth cones exposed to the myosin II ATPase inhibitor blebbistatin and residual retrograde flow can largely be accounted for by the force of leading edge polymerization [3].

Filopodia and veil actin are continuously assembled and transported rearward by retrograde flow. The half-life of growth cone veil actin has been estimated to be 1–3 min whereas filopodial actin bundles are considerably more stable [4] and their recycling requires non-muscle myosin II [3]. To maintain the growth cone periphery in a steady state both of these actin structures must be continuously recycled. Despite the potential mechanistic importance of filament recycling in regulation of growth cone motility, very little is known about the kinetics or spatial characteristics of this processes.

In the current study we use jasplakinolide, a cyclic depsipeptide from the marine sponge *Jaspis johnstoni*, to inhibit network turnover and investigate acute effects on actin filament structure and dynamic behavior. Jasplakinolide cooperatively binds and stabilizes actin filaments by: 1) decreasing the monomer off rate at filament ends and 2) competitively inhibiting cofilin binding and severing activity, resulting in a decrease in the number of uncapped filament ends [5], [6], [7]. Jasplakinolide also increases polymerization rates and promotes nucleation, although this can be largely accounted for by its effect on monomer off rate [5]. Jasplakinolide is commonly used in cell biological experiments to perturb actin dynamics because it is membrane-permeable and has been shown to inhibit protrusion of lamellae, slow cell migration, and induce neurite retraction [8], [9]. Recently, both attractive [10] and repulsive [11] axon guidance cues have been shown to promote actin turnover through activation of ADF/cofilin, however, the cytoskeletal mechanisms underlying these effects are not well understood. Here we report that inhibiting turnover by stabilizing actin filaments stops actin assembly at the leading edge. Simultaneously myosin II contractility moves and compresses the stabilized actin filaments into the central domain. These observations provide a quantitative mechanistic framework for understanding the role of actin turnover in controlling axon growth and guidance.

## Results

### Turnover inhibition results in actin filament accumulation in the transition zone and actin veil depletion at the leading edge

Neuronal growth cones are spatially segregated into two domains: 1) a peripheral (P) domain characterized by dense actin filament networks and occasional microtubules and 2) a central (C) domain densely populated by microtubules and organelles [12], [13] and visualized here by platinum/palladium replica electron microscopy (EM) (Fig. 1A). The peripheral actin is organized into filopodium and actin veil structures, which form the basis of an actin assembly-driven protrusion module [14]. A transition zone (T) exists at the interface between the peripheral and central domains, characterized by bending and buckling of the proximal roots of filopodia [3] which are comprised of 10–15 actin filaments in polarized bundles [15].

To investigate effects of actin turnover inhibition on growth cone ultrastructure, EM was performed on cells treated with vehicle (Fig. 1B) or 500 nM jasplakinolide, a concentration known to inhibit actin filament depolymerization in a pyrene-actin assay [5], for six minutes (Fig. 1C). A striking result was buildup of buckled filopodial actin bundles (Fig. 1B&C, yellow arrowhead) in an expanded transition zone (demarcated by dashed yellow lines). A second major effect was loss of veil actin networks situated between filopodia near the leading edge (Fig. 1, compare control leading edge (B) with area near dashed white line (C)). These ultrastructural changes are consistent with images obtained by fluorescent light microscopy (Fig. S1 A, C).

Growth cones treated for 10 minutes with 70 µM blebbistatin, a specific myosin II inhibitor [16], [17], [18], contained longer, unbuckled filopodia (Fig. 1D) whose proximal roots projected deep into the central domain as predicted from previous light microscopy investigations [3]. Absence of filopodium root buckling in blebbistatin made it difficult to identify the transition zone as a structurally distinct domain. Loss of veil actin did not occur after inhibition of myosin II activity alone (Fig. 1, compare C and E).

Interestingly, growth cones pre-treated with blebbistatin and then exposed to jasplakinolide with continuous myosin II inhibition showed little depletion of veil actin from the leading edge; however, these growth cones still had a distinct buildup of actin filaments in more central regions when compared to growth cones treated with blebbistatin alone (Fig. 1, compare D&E). Together these results suggest actin turnover plays a role in regulating actin network density in both peripheral and central regions of the growth cone and evidence of these processes can be observed in the absence of myosin II activity. We next investigated the actin filament dynamics underlying these structural changes.

### Deceleration of retrograde flow and leading edge retraction follow Jasplakinolide treatment

**Figure 2 pone-0030959-g002:**
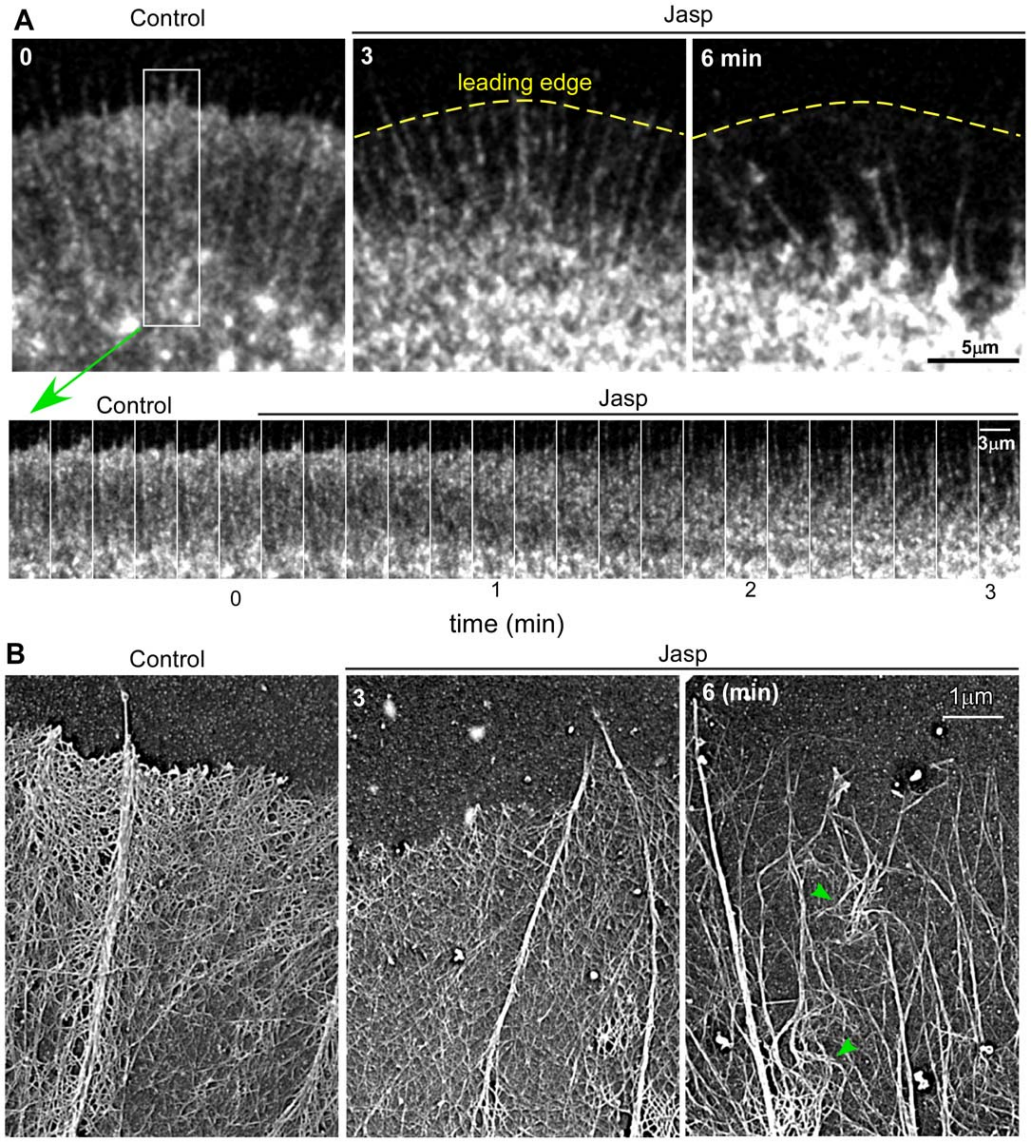
Jasplakinolide results in depletion of actin networks from the leading edge. (A) A bag cell neuron was injected with Alexa-568 actin monomers and imaged using time-lapse, SDC microscopy before and during drug treatment with 500 nM jasplakinolide (100×, 1.4NA objective). Note actin speckle depletion from the leading edge after 6 min in drug (top panel). Bottom panel is a 3 minute duration time montage sampled from the box in (A). (B) Rotary shadowed electron micrographs showing representative leading edge structures under control conditions and after 3 and 6 minutes of jasplakinolide treatment. Note depletion of leading edge veil actin and buckling of filopodia at the 6 min time point.

Clearance of actin from the leading edge and increased actin filament density in the transition zone following jasplakinolide treatment suggested the continued retrograde transport of a population of stabilized actin filaments. To quantitatively address jasplakinolide effects on actin filament dynamics we used fluorescent speckle microscopy [19]. *Aplysia californica* BCNs were injected with a probe for actin, either fluorescently tagged phalloidin or actin monomer, and imaged for ∼5 minutes prior to bath application of jasplakinolide as above. Growth cones were then imaged for 6 or more minutes following jasplakinolide addition depending on the experiment. Within ∼90 sec of jasplakinolide addition a decrease in actin speckle density was evident near the leading edge (time montage 2A, lower) and was striking after 6 minutes (Fig. 2A, upper). EM replicas obtained at the same time points indicated a dramatic decrease in the density of actin veil networks and marked kinking of remaining filopodial actin bundles (Fig. 2B green arrow heads).

**Figure 3:**
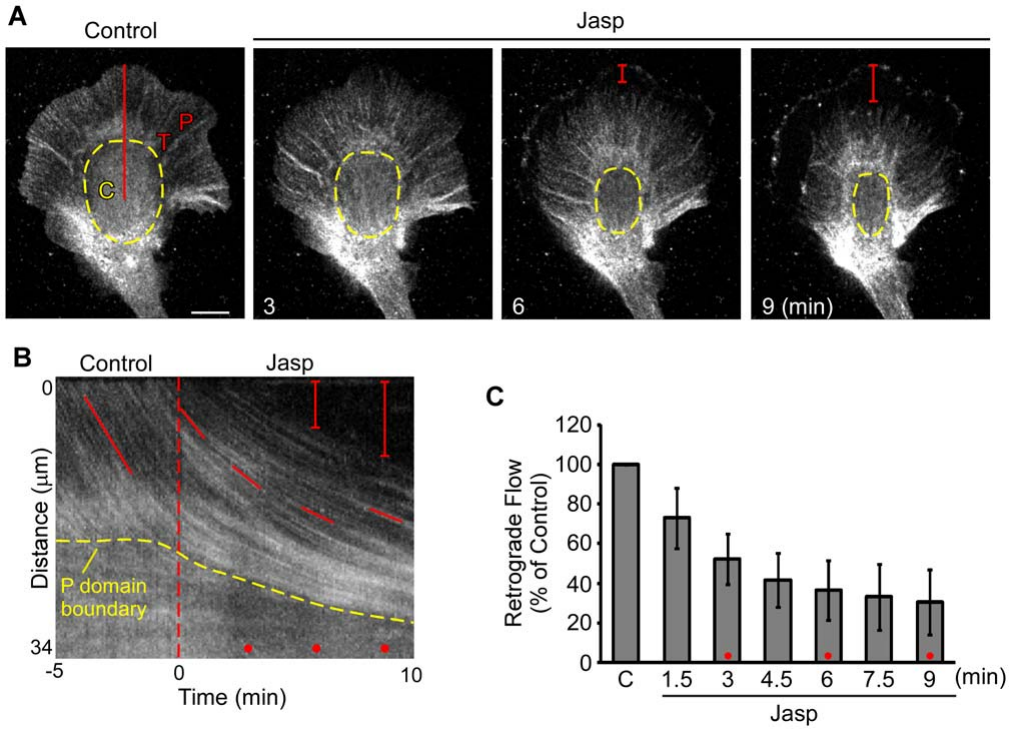
Retrograde flow attenuation accompanies network retraction from the leading edge. Actin images acquired as in Figure 2. (A) Images of the growth cone before, 3, 6, and 9 minutes after bath addition of 500 nM jasplakinolide. The peripheral domain, transition zone, and central domains are marked with P, T, and C respectively. Degree of retraction is indicated by the red brackets in the 6 and 9 minute time-points. The inner border of the peripheral domain and transition zone is delineated by the dashed yellow line. (B) Kymograph taken along the red line in A. The dashed red line indicates the time-point of drug application. The red dots indicate the 3, 6 and 9 minute time points. The red brackets and dashed yellow lines are as in A. The angle of the solid red lines can be used to determine the averaged flow rates over periods of time. (C) Quantification of retrograde flow rates during the time-course of 500 nM jasplakinolide treatment (n = 5: 4 actin and 1 phalloidin). Retrograde flow rates were evaluated within 1.5 minute intervals, normalized verses control and averaged across growth cones. Error bars are standard deviations. Flow rates were 52.4+/−12.7% of control at 3 minutes, and 30.3+/−16.5% at 9 minutes post-treatment. Error bars are standard deviations. Scale bar = 10 um.

Neurite retraction has been reported in response to jasplakinolide [9], however, our use of highly adhesive poly-L-lysine sub strates tended to prevent the membrane leading edge from de-adhering from the substrate. The result was typically a depletion of peripheral actin networks from the adherent membrane edge over time (as in Fig. 2). In addition, central domains of jasplakinolide-treated growth cones appeared to contract and undergo compression. This is shown in the time series in Fig. 3A and supplementary Video S1 (dashed yellow lines approximate central domain area). Kymographs sampled across peripheral and central domains suggested that build up of material in the central domain resulted from influx of peripheral domain actin structures (Fig. 3B, dashed yellow line; cf. Fig. 1C).

Jasplakinolide treatment dramatically reduced peripheral retrograde flow rates (Fig. 3B&C, 4A&B). Rates were assessed by kymography using 1.5 minute sampling windows for a total of nine minutes following jasplakinolide addition. An example kymograph sampled along the red line in panel A is shown in Fig. 3B (dashed red line indicates time of drug application). For population analysis, flow rates (red line slopes) were normalized to the flow rate measured just before drug treatment. Within 3 minutes of jasplakinolide addition retrograde flow had decreased by ∼50% and by 9 minutes flow had further decreased to ∼30% of control levels (Fig. 3C).

**Figure 4:**
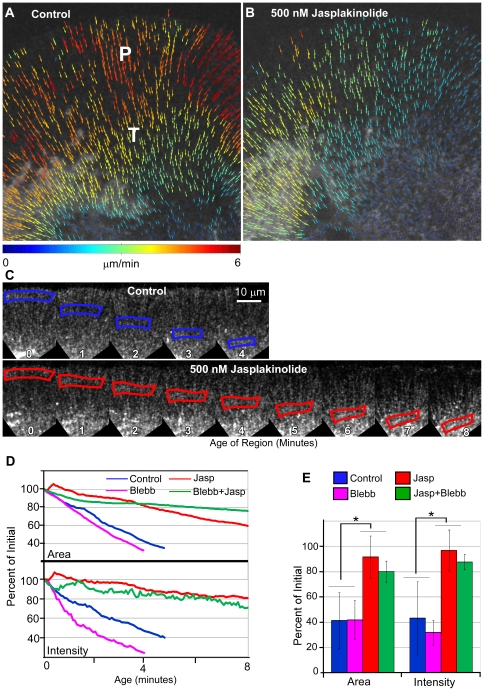
Jasplakinolide inhibits actin filament depolymerization and reduces convergence in the actin network independently of myosin II. (A&B) Vector maps display the spatially averaged speckle displacement between single frame-pairs during the control period (A) and 3 minutes after 500 nM jasplakinolide addition (B). (C) Time montage of flow displaced regions during control (blue border) and 500 nM jasplakinolide treatment (red border). Age 0 for the jasplakinolide region was defined 25 seconds after drug application. The regions tracked in C are from the same growth cone analyzed in A&B. (D) Plot of change in area (top) and change in intensity (bottom) detected within the regions tracked in (C). Blue lines: control; red lines: jasplakinolide. Green lines: blebbistatin+jasplakinolide. Magenta lines: blebb. (E) Pooled data for area and intensity changes during control or drug treatments, Control: 10 regions tracked in 3 growth cones; Jasp: 7 regions from 2 growth cones. Each region was tracked over a 3 minute period during the control and beginning 90 seconds after jasplakinolide treatment. The area and total intensity in each frame was normalized to the initial value at time 0 and graphed as percent of initial values; error bars represent the standard deviation of all 6 regions. Control area and intensities were: 41.29+/−22.25% and 43.88+/−28.8%, respectively. In jasplakinolide area and intensities were: 91.66+/−16% and 96.9+/−16.2% respectively. In blebbistatin, area and intensities were: 41.9+/−15.5% and 31.9+/−9.9%, respectively. In blebbistatin plus jasplakinolide area and intensities were: 80.2+/−8.4% and 87.8+/−6.2%, respectively. Error bars are standard deviations. (*: significance level p<0.001 using a two sample Student's t test, unequal variance).

### Jasplakinolide treatment inhibits actin depolymerization and reduces network convergence independent of myosin II activity

Our results suggested jasplakinolide was simultaneously reducing actin density in the peripheral domain and increasing actin density in the central domain. To address the underlying mechanism, we developed a custom algorithm to measure changes in actin network density and estimate net actin turnover dynamics in defined regions of interest over time (see Material and Methods and Fig. S2 for details). Briefly, the user specifies a polygonal region of interest (ROI) where actin network density changes will be assessed, then actin particle trajectories [20] are used to displace the ROI over time. Assuming spatially smooth flow fields, changes in the integrated actin intensity within the region reflect the net contribution of polymerization and depolymerization. If polymerization and depolymerization were perfectly balanced, no change in integrated intensity would be expected regardless of change in area.

Flow fields from single particle tracking show centripetal movement of actin filaments, with all vectors pointing towards the center of the growth cone (Fig. 4A). Consistent with previous reports, we observed faster flow in the periphery and slower flow in the transition zone and central domain, under control conditions [21], [22]. Following jasplakinolide treatment P domain retrograde flow rate decreased (Fig. 4B), consistent with the kymograph results.

The region tracking algorithm described above was then used to measure changes in actin filament density and network convergence before and after jasplakinolide treatment. Examples of tracked ROIs are shown in Fig. 4C and Video S2. Changes in ROI area and intensity are plotted in Fig. 4D. Over the five minutes the control ROI was tracked, the integrated intensity steadily decreased to ∼40% of its initial value (blue lines). The near constant slope of this curve indicates that depolymerization occurs throughout the peripheral domain. In marked contrast, the intensity within the tracked ROI remained high, retaining 80% of its initial value after 8 minutes in jasplakinolide (500 nM). The same trends are evident in pooled data (Fig. 4E, see legend for statistical details).

Inhibition of actin filament turnover also had a dramatic effect on flow convergence (Fig. 4, C–E, Video S2). In the example shown (Fig. 4C), the control ROI area decreased by 64.7% over 5 minutes, whereas, ROI area decreased by only 38.5% after 8 minutes in jasplakinolide (Fig. 4D, top). The average decrease in area for all ROIs tracked under control conditions was 59.7+/−22.2% of initial levels after 3 minutes; the decrease in area after 3 minutes in jasplakinolide was only 8.3+/−16.7% (Fig. 4E).

We previously reported a role for non-muscle myosin II in severing of actin bundles associated with filopodium roots in growth cones [3] and myosin II has recently been implicated in promoting actin network turnover in migrating keratinocytes [23]. Surprisingly, we found that myosin inhibition with blebbistatin, a specific non-muscle myosin II inhibitor [16], [17], [18] we have characterized in our system [3], had no significant effect on either turnover rates or convergence of actin networks in the P domain (Fig. 4D–E).

### Actin filament turnover and myosin II contractility independently contribute to retrograde actin network flow

**Figure 5:**
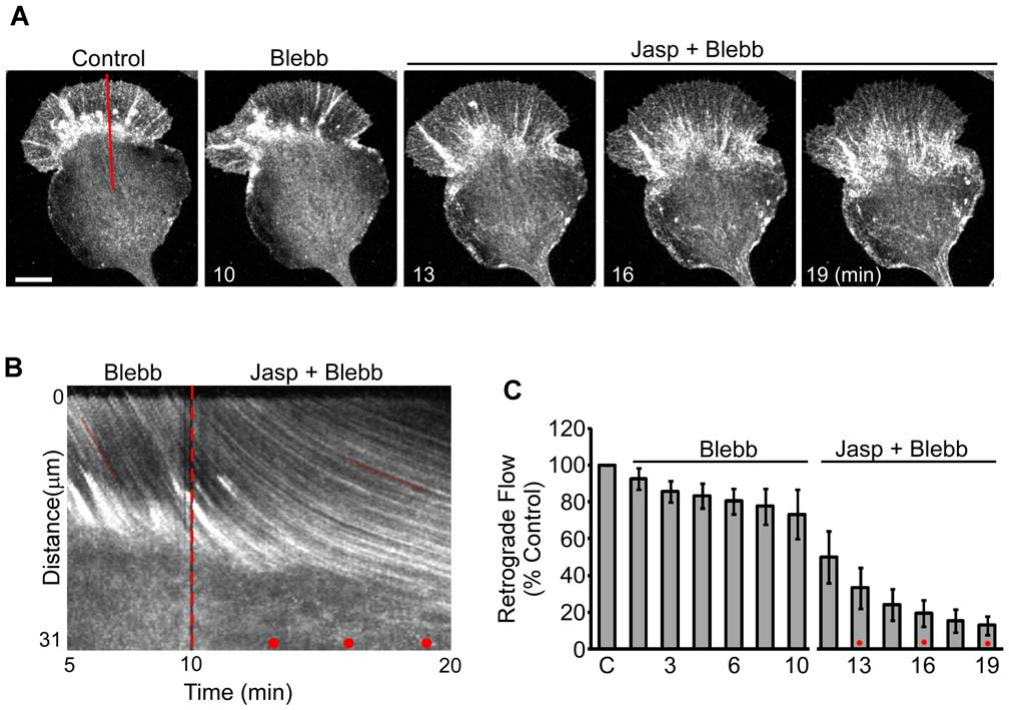
Myosin II inhibition prevents growth cone collapse but not retrograde flow attenuation by Jasplakinolide. (A) Actin network as visualized by SDC-FSM using Alexa-568 actin after 5 minutes in vehicle (0.75% DMSO, panel 1), 10 minutes in 70 µM blebbistatin (panel 2) and 3, 6, and 9 minutes in 70 µM blebbistatin and 500 nM jasplakinolide (panels 3–5 respectively). The peripheral domain, transition zone and central domains are indicated by P, T, and C respectively. The solid red line in the control indicates where the kymograph in (B) was taken. (B) A kymograph of the peripheral domain and transition zone taken along the solid red line in (A). The time period shown includes 5 minutes in blebbistatin alone and 10 minutes in blebbistatin and jasplakinolide. The dashed red line indicates the time of jasplakinolide addition; the red dots indicate 3, 6, and 9 minutes post addition. Notice that no retraction of the leading edge is seen in A or B. (B) As previously reported, blebbistatin pretreatment inhibited retrograde flow. (C) Quantification of retrograde flow rates during the time-course of treatment (n = 12: 7 actin and 5 phalloidin). Retrograde flow rates were evaluated within 1.5 minute intervals, normalized verses control and averaged across growth cones. Normalization and averaging is justified by the strong correlation between initial and final flow rates (0.798, calculated in Excel). Flow rates were 73.4+/−13.3 percent of control after 10 minutes in blebbistatin, 33.2+/−11.1 percent of control at 3 minutes, and 12.8+/−5.2 percent of control after 9 minutes in blebbistatin and jasplakinolide. Error bars are standard deviations. Scale bar = 10 um.

To parse out myosin II's role in jasplakinolide effects on retrograde flow, cells were pretreated with blebbistatin (70 µM, 10 min) and then exposed to jasplakinolide (500 nM) in the continued presence of blebbistatin (Fig. 5). Retrograde flow rates were assessed by kymography every 1.5 minutes under control conditions, then in blebbistatin, and finally, in blebbistatin plus jasplakinolide (Fig. 5B–C). Pretreatment with blebbistatin for 10 min decreased retrograde flow rates to about 75% of control levels and prevented retraction of actin networks from the leading edge seen with jasplakinolide (cf. Fig. 1–2 vs. Fig. 5A–B, Video S3). Addition of Jasplakinolide in the continued presence of blebbistatin reduced retrograde flow rates further to 12.8+/−5.2% of control levels within 9 min (Fig. 5B–C). Residual network flow in blebbistatin plus jasplakinolide (Figure 5C) was significantly slower than that reported in Figure 3 for jasplakinolide alone (p<0.01; time-matched two-tailed Student's t-test).

**Figure 6:**
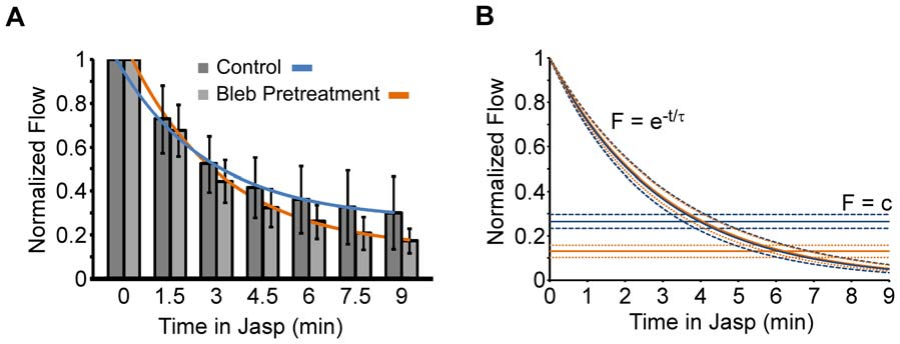
Decay of flow rate with time following turnover inhibition is independent of myosin II. (A) Retrograde flow rates for vehicle control and blebbistatin pretreated growth cones normalized to rates assessed just before jasplakinolide addition. Bar graph shows average flow rates assessed by kymography. Error bars are standard deviations. Solid blue and orange lines are the curves fit to Equation 1. The adjusted R^2^ values of the fit are 0.9982 and 0.9991 for vehicle and blebbistatin pretreated data respectively. (B) Theoretical fit of flow decay timecourse separating the exponential decay from the offset. Solid lines indicate the curve according to the best fit parameter; dashed lines were calculated with the 95% confidence intervals which were between 2.66 and 3.40 min for vehicle and 2.82 and 3.34 min for blebbistatin pretreatment. Blue lines: control data; orange lines: blebbistatin pretreatment data.

Aside from the negative offset introduced by blebbistatin pretreatment, we noted that the kinetics of jasplakinolide induced actin flow decay in blebbistatin were quite similar to those observed after jasplakinolide treatment alone. These results suggested the drug effects could be due to two separate additive processes. In agreement, the decay of retrograde flow was well fit by a first order exponential process with an offset (Equation 1, Materials and Methods) when flow rates were normalized to levels measured immediately before jasplakinolide addition (Fig. 6A). The adjusted R^2^ value with or without blebbistatin pretreatment were 0.9982 and 0.9991 respectively. The decay time constant τ derived from the fit of the averaged jasplakinolide alone data was 2.99 minutes. The decay constant derived from the blebbistatin pretreatment data set was 3.01 minutes (Fig. 6B). The nearly identical decay time constants measured in the presence or absence of blebbistatin suggests jasplakinolide attenuates retrograde flow by a mechanism that is independent of myosin II activity. In contrast, the retrograde flow offset was significantly decreased by blebbistatin treatment- consistent with myosin II activity setting the baseline retrograde flow rate as previously reported [3].

Jasplakinolide effects on retrograde flow cannot be attributed to displacement of myosin II from the actin network since myosin II localization in jasplakinolide treated growth cones was similar to that observed in controls (Fig. S1).

### Turnover inhibition reduces the density of peripheral barbed ends

Jasplakinolide treatment decreased the rate of retrograde network flow in the P domain (Figs. 3A–B); but despite the slower rate of actin filament transport away from the leading edge, actin filament density here was dramatically reduced (Fig. 1C). These observations suggest jasplakinolide was reducing the rate of actin assembly which depends on the concentration of polymerization competent actin monomers and the number of uncapped barbed ends [24]. Barbed ends can be generated both through *de novo* filament nucleation and severing of existing actin filaments. To further investigate which processes were involved we designed experiments to assess changes in barbed end density and measure actin monomer concentration.

**Figure 7:**
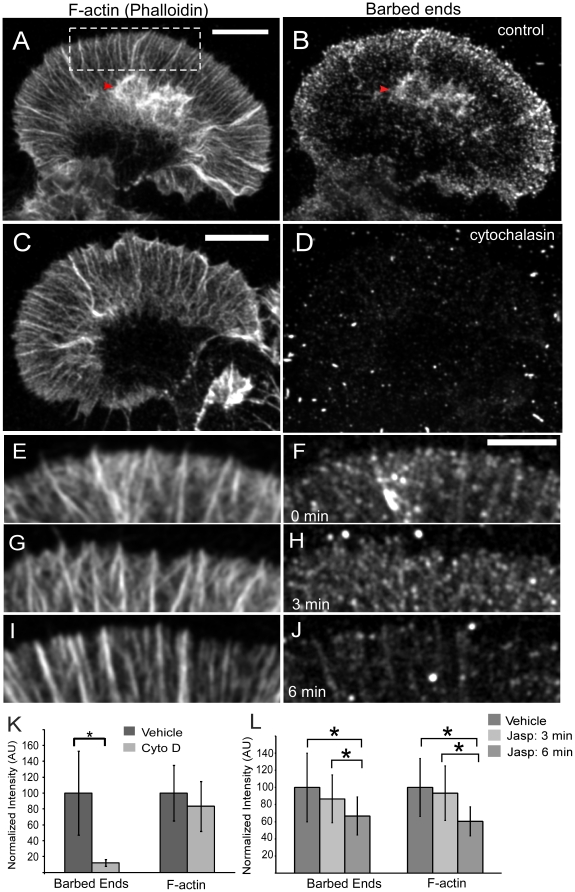
Jasplakinolide treatment reduces barbed end density at the leading edge. Growth cones were dual-labeled with TRITC-phalloidin to show total filamentous actin (A, C, E, G, I) and Alexa 488 g-actin, which incorporates at barbed ends (B, D, F, H, J). (A–B) Barbed ends are concentrated at the leading edge of the growth cone peripheral domain and with intrapodia (red arrowhead) under control conditions. G-actin incorporation is eliminated in the presence of 5 uM cytochalasin D (D&K), while total F-actin is unaffected (C&K), demonstrating the specificity of this labeling procedure for uncapped barbed ends. 6 min treatment with jasplakinolide reduced barbed end density relative to control (L, compare J and F). After 6 min the change in barbed end density was significantly different from both control and 3 min time points (L, compare F&H vs J). A similar pattern was seen for total filamentous actin (L, compare E&G vs I). K) Control: 40 growth cones, CytoD: 32 growth cones. L) Control: 32 growth cones, Jasp: 39 growth cones, Jasp+bleb: 43 growth cones. Examples in E–J are regions similar in size to the dashed white box in A. Error bars are standard deviations. (*: significance level p<0.05 using a two sample Student's t test, unequal variance). Scale bars: A&C 10 µm; F 5 µm.

To measure jasplakinolide effects on barbed end density, cells were permeabilized and existing actin networks stabilized TRITC-phalloidin. Cells were then incubated with Alexa-488-G-actin just above the critical concentration for assembly in the presence of ATP to allow barbed end visualization [25]. Under control conditions barbed ends were concentrated in a ∼2–3 um band at the leading edge and proximally in the transition zone (Fig. 7a and 7b) where they appeared to be associated with intrapodia (red arrowheads; [26]). Cytochalasin D, a small molecule that inhibits actin polymerization by capping barbed ends, prevented monomer incorporation confirming the specificity of this labeling protocol (Fig. 7C, D, and K).

Studies support a role for cofilin-mediated severing of actin filaments and subsequent disassembly in maintaining a pool of G-actin in cells required for stimulus induced actin nucleation at the leading edge [27], [28]. Local severing to create free barbed ends may also regulate assembly at the leading edge [29], [30], [31]. Since jasplakinolide inhibits cofilin-mediated severing, we used the G-actin incorporation assay described above to investigate jasplakinolide effects on barbed end density near the growth cone leading edge. Barbed end density (Fig. 7F, H, J and L) as well as phalloidin labeling at the leading edge (Fig. 7E, G, I and L) progressively decreased over time with exposure to 500 nM jasplakinolide. Jasplakinolide competes with phalloidin binding [32] so fluorescent phalloidin quantification may underestimate F-actin density. However, the presence of phalloidin in the extraction buffer should not affect the density of pre-existing barbed ends being assayed. The above observations are also in agreement with the ultrastructural and FSM data presented in Fig. 1–3 that show a decrease in veil actin filament density near the leading edge after jasplakinolide treatment. Our results suggest that the pool of polymerization-competent barbed ends is sustained by ongoing actin turnover.

### Actin turnover elevates actin monomer concentration in the peripheral domain

The hypothesis that monomer concentration limits polymerization at the leading edge is supported by theoretical models, which predict gradients of actin monomer in the cell. The shape of the G-actin gradient varies according to the location and magnitude of monomer sources (depolymerization sites) and sinks (polymerization sites); however, simulations to date predict lowest G-actin monomer concentration at the leading edge and a ∼1.5-fold increase from front to rear [33].

<1?tlsb=.012w?>We hypothesized that a decrease in G-actin concentration might contribute to the reduced rates of actin assembly after jasplakinolide treatment; however to our knowledge, quantitative assessment of actin monomer concentration gradients corrected for path-length errors has never been reported in growth cones.

To address this problem, growth cones were stained with OG-488 DNAse1, which reliably reports changes in G-actin content in cells [34]. Cells were treated with vehicle (0.005% DMSO) or jasplakinolide (500 nM) for six minutes prior to visualization of G-actin levels with OG-488 DNAseI. To correct for optical path-length differences and allow calculation of relative G-actin concentrations, neurons were injected with 10 kD Texas-Red lysine-substituted dextran, an inert cytoplasmic volume marker that can be fixed in place by aldehyde cross-linking. The process of fixation and membrane extraction reduced the absolute intensity of fixed dextran by 17%; however, the measured volume profile of the growth cone was unchanged (Fig. S3).

**Figure 8:**
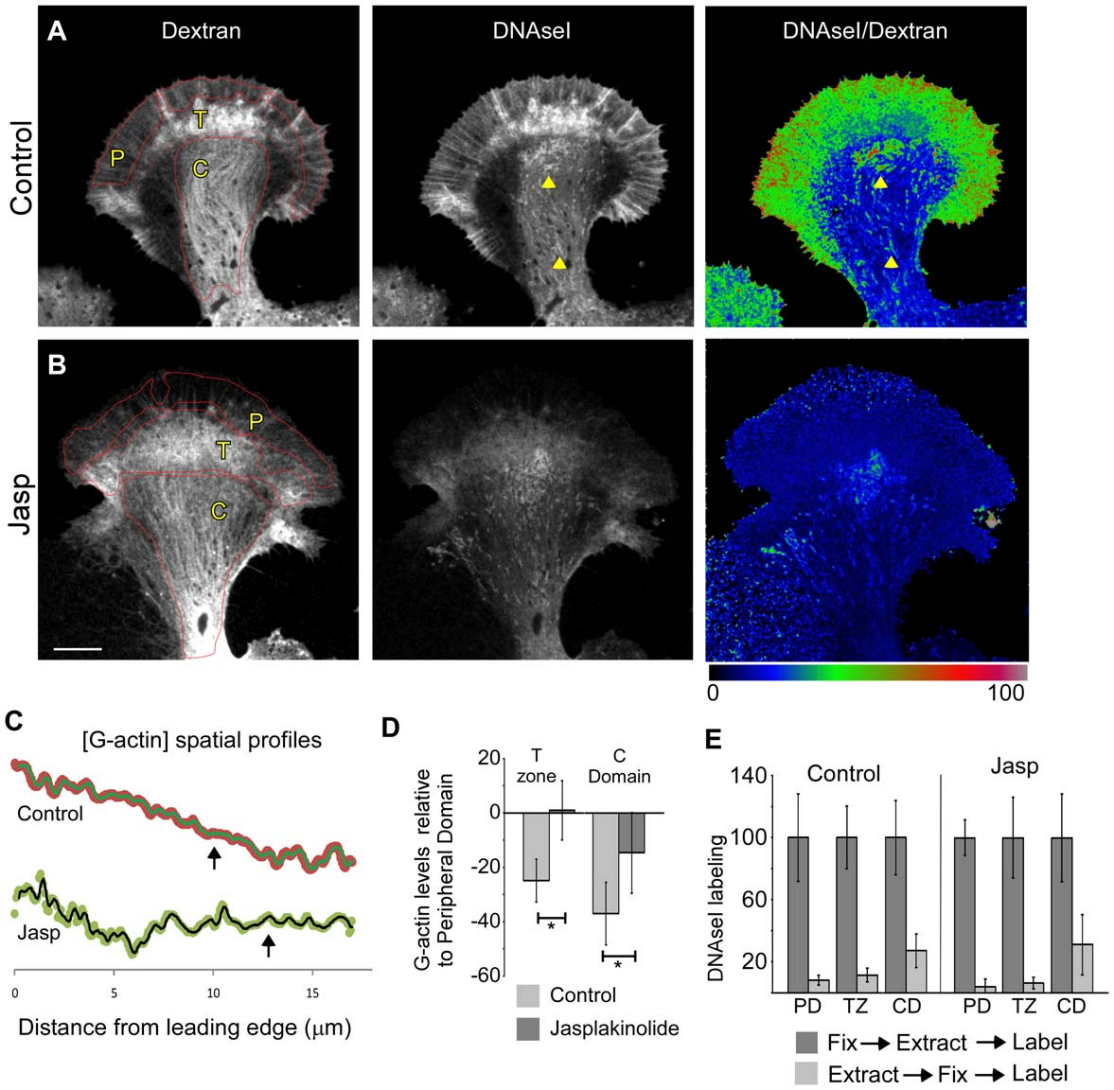
Actin turnover is required to maintain an enhanced concentration of actin monomers in the peripheral domain. A) Cytoplasmic volume detected using injected, lysine-fixable, 10 kD TxRed-dextran (left); actin monomers detected by OG488-DNAseI (middle), and actin monomer concentration estimated by the DNAseI/Dextran (i.e. G-actin/volume) ratio in cells treated for 6 minutes with vehicle (0.05% DMSO, panel A) or 500 nM jasplakinolide (panel B). Red outlines: P = peripheral domain, T = transition zone, and C = central domain areas used for quantification. Arrowheads in DNAseI images indicate mitochondria found in C domain. DNAse/Dextran ratios were normalized by histogram sliding and stretching and a linear color lookup table used for display. C) Spatial profiles of G-actin concentration in Control vs Jasplakinolide treated growth cones in panels A–B. Plots are 4 point rolling averages of line scan intensities sampled from the leading edge into the C domain. Transition zone position is indicated by arrows (see Methods for details). D) Average changes in actin monomer concentration in the transition zone or central domain relative to the P domain in the absence and presence of jasplakinolide. Error bars are standard deviations, N = 12 and 13 growth cones in control and drug treatment conditions respectively. * = P<0.001 (E) Analysis of DNAseI G-actin probe solubility. Neurons were treated for 6 minutes in vehicle or 500 nM jasplakinolide, then DNAseI labeling assessed in cells that were fixed first then permeabilized or in live cells permeabilized in a cytoskeletal stabilization buffer and then fixed [34] (Note that most of the DNAseI in the P domain is soluble; see Methods). Labeling was normalized by total DNAaseI levels assessed in cells fixed before permeabilization. Error bars are standard deviations; n = 8 (control, fix→extract→label), 12 (control, extract→fix→label), 11 (drug, fix→extract→label), and 12 (drug, extract→fix→label). Scale bar = 10 um.

The intensity profiles of cytoplasmic volume and DNAseI were remarkably similar (Fig. 8A, compare *dextran* and *DNAseI* panels) suggesting that most of the DNAse1 intensity variation was due to differences in path-length (volume). However, when the DNAseI signal was divided by the dextran (volume) signal, a marked concentration gradient was revealed under control conditions (Fig. 8A, panel 3; intensity profile 8C). Specifically, the average concentration of actin monomer was significantly lower in the transition zone and central domain than in the peripheral domain (Fig. 8C). Within the overall inward facing negative gradient trend, a variety of G-actin concentration profile shapes in individual growth cones was noted (Fig. S4).

The monomer gradient collapsed in growth cones treated with jasplakinolide showing that continuous turnover of actin filaments is required for maintenance of the gradient (Fig. 8B–C). These results suggest that in addition to reducing barbed end density, inhibition of filament turnover slows actin filament assembly by lowering monomer concentration in the peripheral domain.

Two artifacts associated with the use of DNAseI to label actin monomers may affect the relative concentrations measured here. In addition to binding actin monomers, DNAseI also binds actin filament pointed ends [34], [35], [36]. Since the peripheral domain and transition zone contains a higher density of actin filaments and pointed ends than the central domain, DNAseI labeling intensity would be expected to be higher here and the actin monomer concentration overestimated. To investigate the magnitude of this artifact, we performed live cell extractions using Triton X-100 to permeabilize cells and remove all soluble G-actin before measuring residual DNAseI binding. DNAseI signals from the permeabilized growth cones were normalized by levels obtained from control growth cones that were fixed before permeabilization and DNAseI labeling (Fig. 8D).

DNAaseI labeling in the live cell extracted growth cones was actually lowest in the peripheral domain and transition zone where pointed end density would be expected to be high and highest in the central domain which has lower actin filament density. These results suggest DNAseI labeled G-actin is highly extractable in peripheral regions where actin filament density is high. Lower DNAseI extractability in the central domain is in part due to the presence of mitochondria in this region which also bind DNAseI (Fig. 8 A–B, red arrowheads). Note that mitochondria also tend to exclude cytoplasmic volume; thus, their presence in the central domain would have the net effect of raising the measured DNAseI/Dextran signal and overestimation of actual G-actin concentrations in the central domain. Taking the above into consideration, the DNAseI/Dextran intensity map shown in Fig. 8A is likely an underestimation of the actual G-actin concentration gradient between the peripheral and central domains.

## Discussion

### Depolymerization is a distributed process in the peripheral domain

Actin network density is highest near the leading edge and markedly lower in proximal regions near the transition zone (Fig. 1A). We found that actin density within an ROI steadily decreased as the ROI moved centripetally with retrograde flow despite net ROI compression due to convergence of network flow. Since our approach measures net turnover, the difference between polymerization and depolymerization, we conclude from the consistent decrease in integrated ROI intensity that depolymerization outweighs polymerization throughout the peripheral domain away from the leading edge.

Our results agree with reports in which actin filament turnover was measured with single particle tracking of actin speckles [37], [38]; however, they contrast with other reports suggesting actin filament turnover is concentrated in the transition zone [23]. We note that the latter study used fluorescent phalloidin as a proxy for polymerized actin. Underestimation of actin filament density near the leading edge may account for this discrepancy since use of phalloidin as an actin probe tends to underestimate the density of newly assembled actin filaments near the leading edge and overestimate the density of older filaments in proximal regions due to the slow binding kinetics of phalloidin [21], [39].

### Coincident Deceleration of Retrograde Flow and Network Retraction

Subsequent to actin turnover inhibition, coincident decreases in retrograde flow and leading edge actin network retraction were observed. Both live cell fluorescent images and electron micrographs show a buildup of actin filaments in the transition zone and central domain following turnover inhibition (Fig. 1C & Fig. 2). Build-up of actin filaments in central regions may progressively physically obstruct network compression and contribute to the observed retrograde flow deceleration. A similar inverse relationship between actin filament movement and network density is seen in simplified *in vitro* actomyosin contractile systems [40], [41].

Fluorescence and EM images show decreased actin filament density near the leading edge -also coincident with retrograde flow deceleration. Quantification of free barbed ends also revealed markedly lower actin assembly site densities near the leading edge after jasplakinolide treatment (Fig. 7). Since polymerization tends to generate pushing forces near the leading edge [42] that can drive retrograde flow in growth cones [3], less net actin assembly could contribute to the observed slower flow rates. Note that actin network retraction from the leading edge is expected whenever net actin assembly rates fall below retrograde flow rates, however, once the network separates from the leading membrane edge it can no longer contribute pushing force to drive retrograde flow [12]. In summary, we suggest that the superimposed effects of: 1) reduced leading edge actin assembly rates and 2) continued central network compression by myosin II dependent forces account for the leading edge retraction we observed following jasplakinolide application.

### What is the role of myosin II in actin network turnover?

Recent studies suggest a role for myosin II in actin network turnover [23], [43], [44], [45]. Here we have analyzed actin movement and turnover in the presence and absence of myosin II activity and found that myosin has little effect on bulk turnover in the peripheral domain. This is shown explicitly in Figure 4 where tracked regions in blebbistatin treated growth cones had similar fluorescence decay profiles to control. The kinetics of retrograde flow decay after turnover inhibition were also little affected by myosin activity. In fact, the effects of blebbistatin and jasplakinolide were mathematically separable with myosin activity contributing a constant offset to the exponential decay of flow (Fig. 6).

Turnover inhibition with jasplakinolide immediately attenuates leading edge polymerization. In contrast, *Aplysia* growth cones can sustain a steady state network treadmill after myosin inhibition with persistent retrograde flow at 50–75% of control rates, as previously reported [3]. This suggests that leading edge actin polymerization can drive the peripheral network treadmill independent of myosin II activity.

We previously reported a specific role for myosin II in filopodium actin bundle severing and recycling in the growth cone transition zone [3]. This effect, which can result in filopodial actin bundle elongation to ∼200% of control lengths, is evident in Fig. 1B vs. D. We therefore suggest that myosin II affects retrograde flow rate and turnover of filopodia, but has little influence on the bulk turnover of the peripheral domain necessary for maintenance of leading edge polymerization.

### How does turnover inhibition affect leading edge polymerization?

Contrary to previous predictions [33], we found higher levels of G-actin monomer in the peripheral domain than in more central regions. In many growth cones a clear inward facing negative concentration gradient of actin monomer was observed (Fig. 8, Fig. S4). Our findings suggest the presence of an active process [46] to maintain the concentration gradient in the face of constant actin filament assembly and consequent monomer depletion near the leading edge. A potential solution would be an anterograde cytoplasmic fluid flow capable of carrying soluble proteins from central regions toward the leading edge similar to what has recently been described in motile epithelial cells [47].

Other reports, both experimental and theoretical, hint that turnover may be required to maintain the monomer pool at the leading edge [8], [33], [34]. In agreement, we observed that the monomer gradient rapidly collapsed following turnover inhibition with jasplakinolide. Our findings suggest that actin filament turnover occurs throughout the peripheral domain (Fig. 4C–D). This process creates a distributed source of actin monomers that in concert with forward fluid flow could account for the high steady state G-actin concentrations we observe in the peripheral domain.

In addition to *de novo* nucleation, actin filament severing generates uncapped barbed ends that can serve as sites for monomer addition [30], [33]. In agreement, we found the highest density of exposed barbed ends near the leading edge (Fig. 7B). Significantly, turnover inhibition rapidly reduced free barbed end density here, possibly as a result of competition between jasplakinolide and cofilin for binding to actin filaments.

We noted that following jasplakinolide treatment actin veil density was diminished relative to actin bundles comprising filopodia (Figs. 1C, 2, and 3A). It has been previously reported that filopodia have a longer half-life than veils [4] and therefore may be more stable structures. We speculate that changes in G-actin concentration could differentially affect veil versus filopodial actin assembly. In particular filopodia may be able to continue to grow processively in jasplakinolide to the extent that they persist after the veil has retracted.

What is the general cell biological relevance of the above? The actin polymerization rate is proportional to the concentrations of uncapped filament ends and polymerization competent actin monomer. Barbed ends are generated by de novo actin nucleation and severing of existing filaments, while actin monomers are generated by severing and depolymerization. Both these processes have been implicated in regulation of cell motility. For example, photoactivation of cofilin in cancer cells induces crawling in the direction of activation [30]; cofilin is also required for EGF-stimulated lamellipodium extension [25], [48]. EGF stimulation increases barbed end density and leading edge actin polymerization in carcinoma cells [49]. Similar results were reported in response to NGF stimulation of neuronal growth cones [10]. Exogenous G-actin monomer can also compensate for cofilin deficiency [28] suggesting that ADF/Cofilin activity maintains a pool of actin monomer for feeding barbed end growth. This extensive body of literature and our current results point to the importance of actin turnover mechanisms in regulation of actin-based structural dynamics and related motility processes.

### Relevance of Actin Turnover for Neuronal Growth Cone Behavior

Previous studies suggest signaling pathways that regulate the stability of actin filaments can have significant effects on growth cone motility and guidance [10], [11], [50], [51], however, mechanisms underlying these effects are not well understood at the level of cytoskeletal dynamics. To maintain the growth cone's actin network treadmill at steady state, net filament assembly and disassembly must be balanced. The current study suggests that modulation of leading edge actin assembly rates via changes in barbed end density and/or actin monomer concentration are powerful mechanisms by which turnover pathways affect actin based growth cone motility and axon guidance. Under these conditions, changes in any parameter will have immediate and global effects on actin structure.

Myosin II has been implicated as a downstream effector in many axon guidance pathways however the mechanistic role non-muscle myosin II and its various isoforms play in these processes is quite complex [52], [53]. In agreement with previous studies, our results show that myosin II activity is required for jasplakinolide-induced actin network retraction, which has been implicated in growth cone retraction responses [9]. We note however, that the related issue of how actin turnover affects development of traction forces involved in turning and substrate dependent growth [54], [55] remains largely unexplored. Our finding that the kinetic effects of inhibition of actin turnover are independent of myosin II activity (Fig. 3) has mechanistic implications for understanding these processes and highlights the importance of quantitative analysis of the action of drugs commonly used to perturb cytoskeletal processes involved in axon growth.

## Materials and Methods

### Cell Culture and Imaging Media

Primary cultures of *A. californica* bag cell neurons on poly-D-lysine coated coverslips were prepared as described by Forscher and Smith [12], with some modification. Briefly, abdominal ganglia were incubated at 22°C for ∼18 hours in L-15 artificial sea water (ASW) (400 mM NaCl, 10 mM KCl, 15 mM HEPES, pH 7.8, 10 mM CaCl, 55 mM MgCl_2_, and Phenol Red) supplemented to 10 mM N-tert-butyl-alpha-phenylnitrone (PBN) and ∼10 mg/mL dispase. Connective tissue was manually removed and the intact bag cell cluster was triturated gently with a bent 20 µL pipette tip to separate the neurons, which were arranged on a poly-D-lysine coated, acid washed coverslip. Cultures were maintained for up to 24 hours in L-15 ASW at 18°C prior to use. Fluorescence imaging was performed in L-15 ASW (without Phenol Red) supplemented to 0.25 mM Vitamin E, 3 mg/mL L-carnosine, and 10 mM PBN. Unless noted, cells were maintained in a 1∶10,000 dilution of fetal bovine serum from plating onward.

### Microinjection

Microinjection was performed as described previously [21]. For phalloidin injections, 75 µL of methanol phalloidin stock were dried down and resuspended in 4 µL of water for a concentration of 124 µM in the needle. Either Alexa-488 or Alexa-594 conjugated phalloidin (Molecular Probes) was used in any given experiment. For actin monomer injection experiments, 0.5 mg/mL aliquots in G-buffer (5 mM Tris, pH 8.1, 0.2 mM CaCl_2_, 0.2 mM dithiothreitol, and 0.2 mM ATP) were thawed on ice and spun at 20,000 k at 4°C for 30 minutes prior to use. Alexa-488 and/or Alexa-568 rabbit skeletal muscle actin (Molecular Probes) was used in experiments. Lysine-substituted, Texas Red 10 kD dextran (Molecular Probes) was used for volume correction of DNAseI stains. After injection, cells were allowed to recover for at least one hour.

### Immunocytochemistry

Cells were incubated in a flow chamber in Fix (4% formalin, 400 mM NaCl, 10 mM KCl, 15 mM HEPES, pH 7.8, 10 mM CaCl, 55 mM MgCl_2_, and 400 mM sucrose) for 20–30 minutes and 1% Triton X-100 in Fix for 30 minutes prior to three washes with PBS-T (0.1% Triton X-100, 137 mM NaCl, 2.7 mM KCl, 10 mM phosphate, pH 7.5). For antibody labeling, cells were blocked for 30 minutes in 5% BSA/PBS-T, incubated with primary antibody for 20–30 minutes in 5% BSA/PBS-T, washed three times in 5% BSA/PBS-T, and incubated for 30 minutes to one hour in secondary antibody diluted in 5% BSA/PBS-T. To label actin monomers, 5 µg/mL Oregon Green DNAseI (Molecular Probes) in 5% BSA/PBS-T was used after blocking. Cells were washed three times in PBS-T and mounted in MOWIOL media (10% MOWIOL, 25% glycerol, 0.2 M Tris pH 8.5, 20 mM n-propyl-galate).

### Live Cell Extraction

Cells were incubated in a flow chamber for three to five minutes in LE (100 mM PIPES, pH 6.9, 10 mM KCl, 100 mM NaCl, 5 mM EGTA, 5 mM MgCl_2_, 4% PEG, average MW = 35,000, and ∼20% sucrose, ∼1000 mOsm/L) supplemented to 1% Triton X-100, ∼10 µM phalloidin, 10 µM Taxol, and ∼10 mg/mL BAPTA. TRITC labeled phalloidin (Sigma) was used to visualize actin filaments. For electron microscopy, LE solution was supplemented to 0.1% glutaraldehyde. Extracted cells were washed once with WLE (80 mM PIPES, pH 6.9, 5 mM EDTA, 5 mM MgCl_2_) and fixed for 20 minutes in 4% formalin/WLE for fluorescence or 5% glutaraldehyde/WLE for electron microscopy. For barbed end labeling experiments extracted cells were incubated in 300 nM Alexa-488-G-Actin (see above) in WLE prior to fixation. If live cell extraction was to be followed by immunocytochemistry, antibody labeling and slide mounting were performed as above.

### Platinum/Palladium Replica Electron Microscopy

Cells were prepared essentially as described by Svitkina and Borisy [56], with some modifications. Cells were live cell extracted and fixed as previously described. Often the fixed cells were stored in a humidity chamber overnight at 4°C. A second fixation in 5% glutaraldehyde in water for 20 minutes was followed by 20 minutes in 0.2% tannic acid, three water washes, 20 minutes in 0.2% uranyl acetate, and three water washes. Graded dehydration was performed in increasing concentrations of ethanol (10-20-40-60-80-100-100-100^*^-100^*^) prior to critical point drying. The 100^*^ indicates that the ethanol was dried with molecular sieves prior to use. Platinum/palladium rotary shadowing was performed at a 45° angle to a thickness of 2 to 2.5 nm followed by carbon shadowing at a 75° angle. Replicas were mounted on carbon/formvar coated EM grids and observed by transmission EM at 80 kV.

### Microscopy

Images were acquired using an Andor Revolution XD spinning disk confocal built on a Nikon TE 2000E inverted microscope with Perfect Focus System, equipped with an Andor iXon^EM+^ 888 camera, and CSU-X1 confocal head (Yokogawa). Trans-illumination was provided by a halogen lamp and controlled by a SmartShutter (Sutter Instrument). Confocal excitation was provided by the Andor Laser Combiner System with three laser lines: 488, 561, and 647 nm. Emission wavelength was controlled by a Sutter LB10W-2800 filter wheel outfitted with bandpass filters from Chroma. Image acquisition and all other peripherals were controlled by iQ software (Andor). A 100×, 1.4NA lens was used during all imaging.

### Quantitative Actin Dynamics

Kymography and automated speckle tracking were used to determine rates of movement from time-lapse microscopy. Automated flow tracking was performed with the fsmCenter suite of custom Matlab (MathWorks) algorithms, produced by the Danuser lab, according to the strategy outlined Ji and Danuser [20]. Briefly, an adaptive multi-frame correlation algorithm [20] was run on every 5 or 10 frames to determine the average flow over 5 to 10 frames. This information was used to initialize a single particle tracking algorithm [37], [57], [58], [59], which was run on every frame in the movie.

### ROI Based Turnover Analysis

A custom Matlab (MathWorks) algorithm was written to measure the net kinetic activity within a user defined region of the growth cone. The algorithm is based on conservation of mass principles. The major steps in the analysis are enumerated here and visually in Fig. S1:

1. User selects a region outside of the cell that will be used to determine the average background intensity. The average background is determined separately for each image in the series and is subtracted from any intensity measurements made, in that image, on a pixel by pixel basis.
2. The user defines a region of interest (ROI) in the cell that will be tracked throughout the image series.
3. The spatially average flow, determined within a box around each vertex of the ROI, is calculated based upon the single particle tracking results. The size of the averaging box is user selectable. In this analysis, box sizes were between 33 and 75 pixels wide, depending upon the speckle/vector density of the dataset. Flow averaging is required for two reasons: flow maps are sparse and flow tracking contains errors. In other words, the vertex is most likely not on a successfully tracked speckle, and even if it was, that track might be wrong.
4. The ROI is displaced and deformed along the averaged vectors determined at each vertex. This mimics a closed system, assuming that all assembled actin moves with retrograde flow; therefore, changes in intensity must be due to the assembly or disassembly of actin filaments.
5. Steps 3 and 4 are repeated for each frame pair in the series in order to generate plots of area and intensity over time. Changes in area reflect network convergence or expansion in two dimensions; changes in the total intensity within the area reflect the shifting balance of polymerization and depolymerization over time.

To evaluate changes in network compression and net kinetics, three regions were tracked per growth cone. Regions were defined during the control period and tracked for three minutes (37 frames at five seconds per frame). The positions of the regions were reset and tracked for another three minutes, starting 90 seconds after the addition of jasplakinolide. Media exchange induces focus shifts in the time-lapse movie. The delay eliminated any intensity shifts from the analysis.

### Regression Analysis of Retrograde Flow Decay

The curve fitting toolbox in Matlab (MathWorks) was used to model the flow rate changes resulting from drug treatments as an exponential process with an offset according to Equation 1:
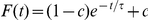
Where F(t) is the flow at time t in minutes, c is the flow offset, and τ is a time constant, defined as the amount of time required for flow to decrease to 1/e of its initial value. The flow rates for individual growth cones were normalized to rates assessed just before jasplakinolide addition. Regression analysis was performed on population averages and individual growth cone results.

### Image Processing

All fluorescent images were subjected to background subtraction prior to quantitative analysis. The lone exception is automated speckle tracking analysis, which was performed upon raw image data. For display only, images were convolved with a Gaussian kernel and scaled according to a linear look up table. For ratio imaging, Gaussian convolution was utilized prior to background subtraction in order to reduce noise levels. A binary mask was used to eliminate noise amplification outside the cell. Ratio images were normalized by sliding and stretching the image histograms and displayed using a linear lookup table. Fluorescent image processing was performed in Metamorph (Universal Imaging), Matlab (MathWorks) or Image J (open source, NIH: http://imagej.nih.gov/ij/index.html) software. Electron micrographs were scaled and sharpened in Photoshop (Adobe) with an 8 pixel radius and between 50 and 100% sharpening.

### Line Scan Analysis

Line scans were used to analyze the spatial intensity distribution of fluorescent probes. Line scans were performed in Metamorph (Universal Imaging) or Image J (NIH) and the data exported to Excel (Microsoft). The growth axis was defined by drawing a line along the presumed axis of growth. Average pixel intensity was sampled using 15–20 pixel width lines were plotted as a function of distance from the leading edge.

## Supporting Information

Figure S1 **Myosin II localization in unaffected by jasplakinolide treatment.** TRITC-Phalloidin stabilized F-actin (A, C) and Myosin II localization (B, D) in growth cones treated for 3 minutes with vehicle (0.05% DMSO, A–B) or 500 nM Jasplakinolide (C–D) and then live-cell extracted. All phalloidin and myosin II images were acquired using the same imaging parameters to enable direct comparison across conditions. (E, F) Line-scan analysis of F-actin (E) and myosin II (F) localization in growth cone. Line scans (20 pixels wide) were sampled in regions indicated by blue and orange lines in A and C respectively and intensities plotted verses distance from the leading edge. Blue diamonds and yellow squares represent individual growth cones sampled under control conditions and in jasplakinolide respectively. Black and red lines represent 20 point rolling averages for control and jasplakinolide treated conditions (n = 27 and 34 growth cones).
(TIF)

Figure S2 **Schematic representation of region tracking algorithm.** Flow displacement and deformation of regions generates a closed system where changes in intensity can be interpreted as reflecting kinetic activity. The principle steps in the algorithm are illustrated here. **Panel 1**) The user is prompted to select a background region (dashed white box outside the cell). The average fluorescence in this box is determined for each frame in the image series and subtracted from each pixel in the frame. **Panel 2**) The user is prompted to select a region of interest to be tracked (Red outline in panel 2). **Panel 3**) The average flow is calculated at each vertex. A user selectable grid size (dashed white box) is centered upon each vertex and all flow vectors detected within the box are averaged. **Panel 4**) The ROI is displaced and deformed along the averaged flow vectors. The dark grey of the ROI interior reflects the idea that this is a closed system.
(TIF)

Figure S3 **Intensity profiles of lysine-substituted dextrans are unchanged during post-fixation membrane extraction.** A bag cell neuron, injected with 10 kD, lysine-fixable, Texas Red dextran (Molecular Probes), was imaged throughout processing for immunocytochemistry. (A–B) Epifluorescence images after 4% formalin fixation only (A) and after 1% Triton X-100 extraction (B). (C) Line scans reporting the average intensity in a 20 pixel wide band along the red and green lines in A and B. The position of the leading edge is indicated by the dashed line. The average background intensity was subtracted from the images prior to the line scan analysis. (D) Ratio of the Fixed line scan divided by the Extracted line scan. Inside the cell, the ratio is flat; however, as the fluorescent signal decreases, the noise increases. The ratio is greater than 1, reflecting a ∼17% loss of signal during 1% Triton X-100 extraction.
(TIF)

Figure S4 **G-actin concentration profiles in control growth cones.** A–D) Four examples of G-actin profiles in growth cones of varying size and shape. Note consistently higher levels in P as compared to C domains despite differences in P domain concentration profile shape. Plots are 4 point rolling averages of intensities sampled from the leading edge into the C domain. Bar = 10 µm.
(TIF)

Video S1 **Actin dynamics during jasplakinolide treatment.** *A. californica* BCN injected with Alexa-568 actin (Molecular Probes) and analyzed by time-lapse spinning disk confocal microscopy (Andor Revolution). Frames were acquired every 5 seconds over the course of 15 minutes. After 5 minutes in vehicle (0.05% DMSO), 500 nM jasplakinolide was added. Scale bar is 10 µm.
(MOV)

Video S2 **Region tracking analysis example.** *A. californica* BCN injected with Alexa-568 actin (Molecular Probes) and analyzed by time-lapse spinning disk confocal microscopy (Andor Revolution). Tracked regions are outlined in red. The left panel shows tracking during vehicle pretreatment; the right panel shows tracking beginning 25 seconds after addition of 500 nM jasplakinolide. Images were acquired every 5 seconds for 5 minutes (vehicle) and 8 minutes 20 seconds (jasplakinolide). Scale bar is 5 µm.
(MOV)

Video S3 **Actin dynamics during blebbistatin and blebbistatin/jasplakinolide treatment.** *A. californica* BCN injected with Alexa-568 actin (Molecular Probes) and analyzed by time-lapse spinning disk confocal microscopy (Andor Revolution). Frames were acquired every 5 seconds for 5 minutes in vehicle (0.75% DMSO), then 10 minutes in 70 µM blebbistatin, followed by 10 minutes in 70 µM blebbistatin+500 nM jasplakinolide. Scale bar is 10 µm.
(MOV)
