# Supplementary figures and images for: The Role of Actin Turnover in Retrograde Actin Network Flow in Neuronal Growth Cones
Source: PLoS One. 2012 Feb 16;7(2):e30959. doi: 10.1371/journal.pone.0030959 (PMC3281045; doi:10.1371/journal.pone.0030959)

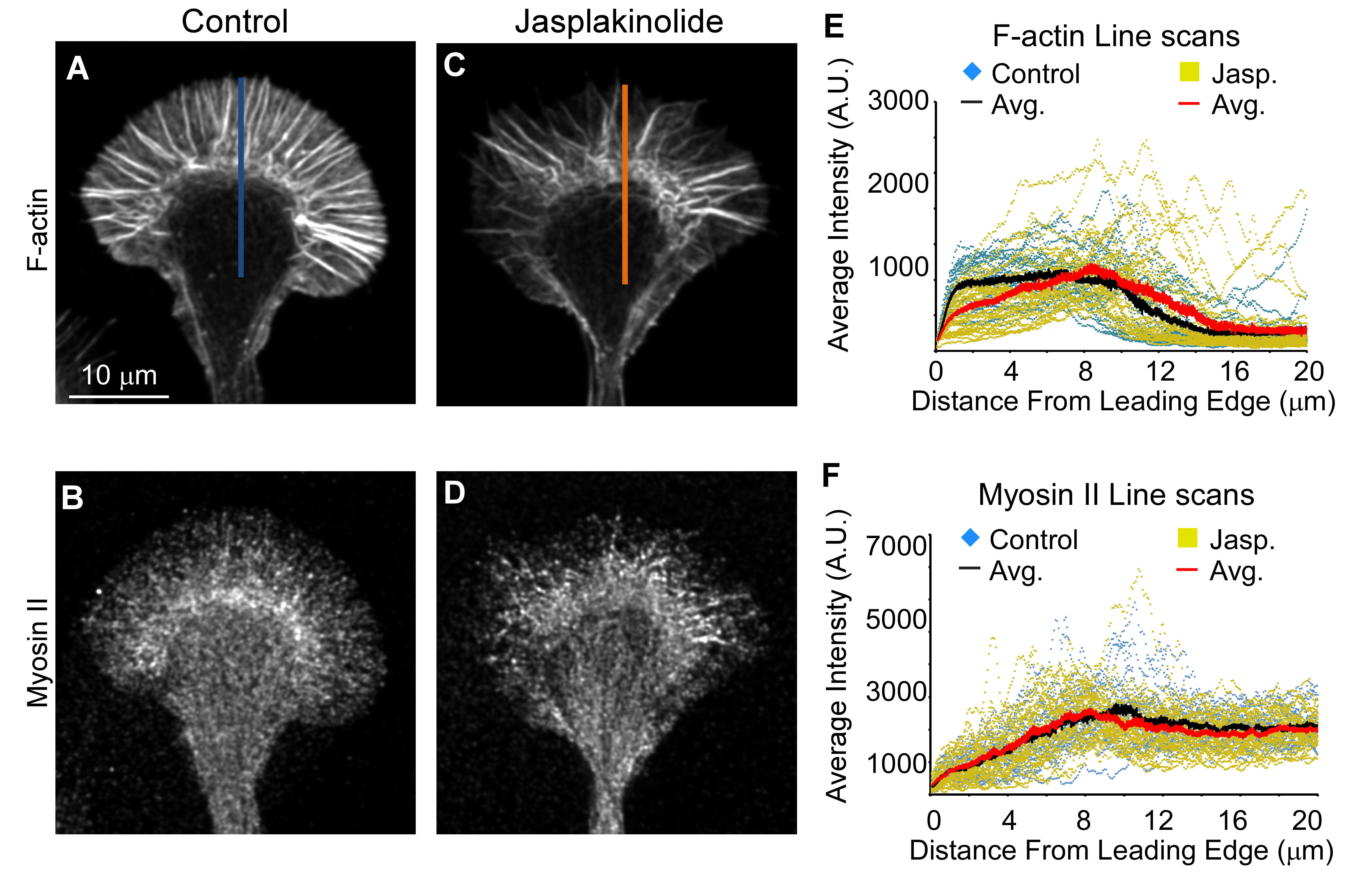

Supplement: Figure S1 — Myosin II localization in unaffected by jasplakinolide treatment. TRITC-Phalloidin stabilized F-actin (A, C) and Myosin II localization (B, D) in growth cones treated for 3 minutes with vehicle (0.05% DMSO, A–B) or 500 nM Jasplakinolide (C–D) and then live-cell extracted. All phalloidin and myosin II images were acquired using the same imaging parameters to enable direct comparison across conditions. (E, F) Line-scan analysis of F-actin (E) and myosin II (F) localization in growth cone. Line scans (20 pixels wide) were sampled in regions indicated by blue and orange lines in A and C respectively and intensities plotted verses distance from the leading edge. Blue diamonds and yellow squares represent individual growth cones sampled under control conditions and in jasplakinolide respectively. Black and red lines represent 20 point rolling averages for control and jasplakinolide treated conditions (n = 27 and 34 growth cones). (TIF) [file pone.0030959.s001.tif]

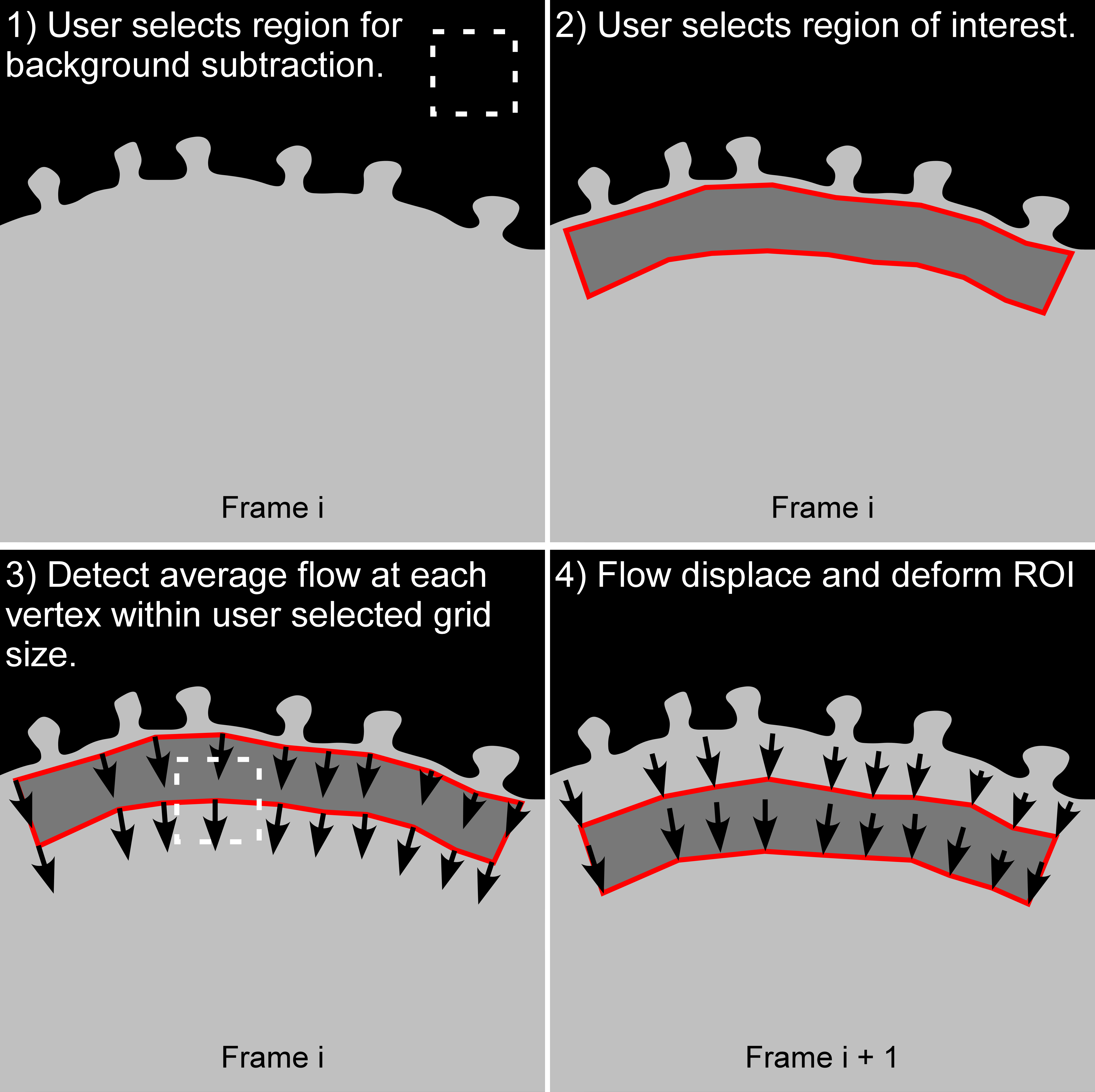

Supplement: Figure S2 — Schematic representation of region tracking algorithm. Flow displacement and deformation of regions generates a closed system where changes in intensity can be interpreted as reflecting kinetic activity. The principle steps in the algorithm are illustrated here. Panel 1) The user is prompted to select a background region (dashed white box outside the cell). The average fluorescence in this box is determined for each frame in the image series and subtracted from each pixel in the frame. Panel 2) The user is prompted to select a region of interest to be tracked (Red outline in panel 2). Panel 3) The average flow is calculated at each vertex. A user selectable grid size (dashed white box) is centered upon each vertex and all flow vectors detected within the box are averaged. Panel 4) The ROI is displaced and deformed along the averaged flow vectors. The dark grey of the ROI interior reflects the idea that this is a closed system. (TIF) [file pone.0030959.s002.tif]

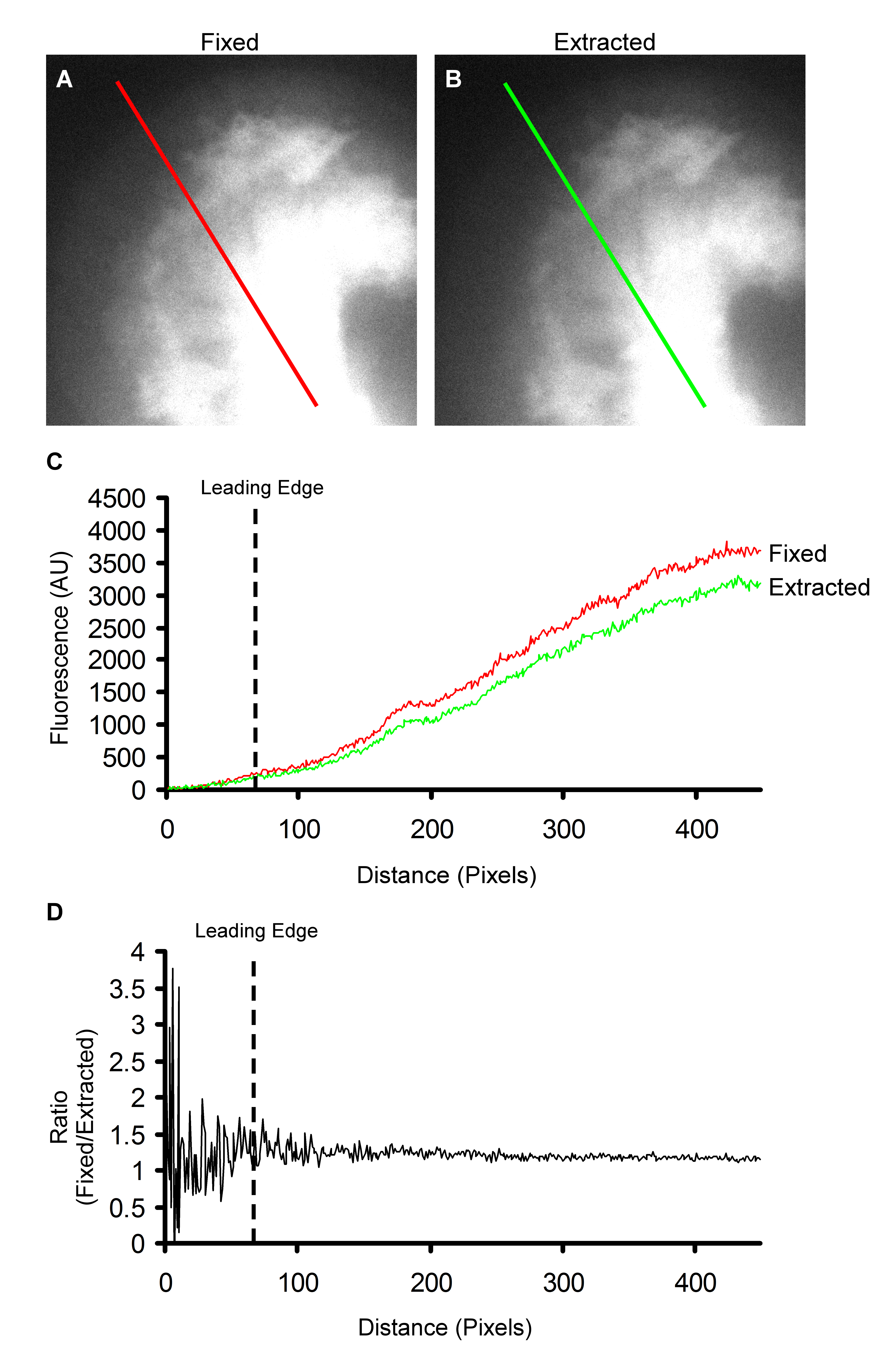

Supplement: Figure S3 — Intensity profiles of lysine-substituted dextrans are unchanged during post-fixation membrane extraction. A bag cell neuron, injected with 10 kD, lysine-fixable, Texas Red dextran (Molecular Probes), was imaged throughout processing for immunocytochemistry. (A–B) Epifluorescence images after 4% formalin fixation only (A) and after 1% Triton X-100 extraction (B). (C) Line scans reporting the average intensity in a 20 pixel wide band along the red and green lines in A and B. The position of the leading edge is indicated by the dashed line. The average background intensity was subtracted from the images prior to the line scan analysis. (D) Ratio of the Fixed line scan divided by the Extracted line scan. Inside the cell, the ratio is flat; however, as the fluorescent signal decreases, the noise increases. The ratio is greater than 1, reflecting a ∼17% loss of signal during 1% Triton X-100 extraction. (TIF) [file pone.0030959.s003.tif]

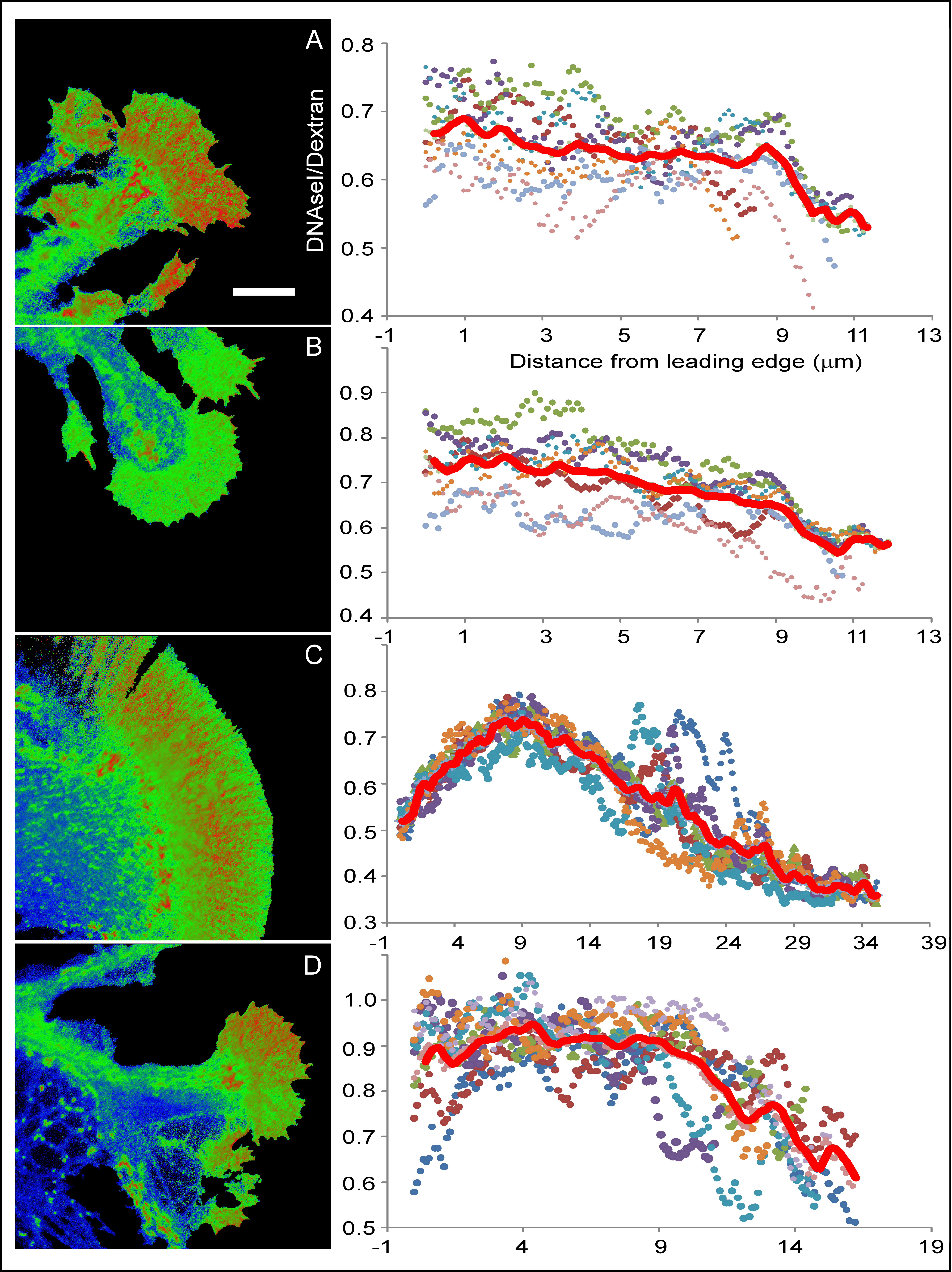

Supplement: Figure S4 — G-actin concentration profiles in control growth cones. A–D) Four examples of G-actin profiles in growth cones of varying size and shape. Note consistently higher levels in P as compared to C domains despite differences in P domain concentration profile shape. Plots are 4 point rolling averages of intensities sampled from the leading edge into the C domain. Bar = 10 µm. (TIF) [file pone.0030959.s004.tif]
